# Improvement of Type 2 Diabetes Mellitus and Attenuation of NAFLD Are Associated with the Success of Obesity Therapy

**DOI:** 10.3390/jcm11071756

**Published:** 2022-03-22

**Authors:** Andreas Schmid, Miriam Arians, Thomas Karrasch, Jörn Pons-Kühnemann, Andreas Schäffler, Martin Roderfeld, Elke Roeb

**Affiliations:** 1 Department of Internal Medicine III, Justus Liebig University, 35392 Giessen, Germany; andreas.schmid@innere.med.uni-giessen.de (A.S.); thomas.karrasch@innere.med.uni-giessen.de (T.K.); andreas.schaeffler@innere.med.uni-giessen.de (A.S.); 2 Department of Gastroenterology, Internal Medicine II, Justus Liebig University, Klinikstr. 33, 35392 Giessen, Germany; arians.miriam@stud.hs-fresenius.de (M.A.); martin.roderfeld@innere.med.uni-giessen.de (M.R.); 3 Institute of Medical Informatics, Justus Liebig University, 35392 Giessen, Germany; joern.pons@informatik.med.uni-giessen.de

**Keywords:** type 2 diabetes mellitus, NAFLD, NFS, liver fibrosis, obesity, bariatric surgery, low-calorie formula diet, HbA1c

## Abstract

Obesity and type 2 diabetes mellitus (T2D) represent important comorbidities of the metabolic syndrome, which are associated with non-alcoholic fatty liver disease (NAFLD)-related hepatic fibrosis. In total, 160 morbidly obese patients—81 following a low-calorie formula diet (LCD) program and 79 undergoing bariatric surgery (Roux-en-Y gastric bypass, RYGB)—were examined for anthropometric and metabolic parameters at base-line and during 12 months of weight loss, focusing on a putative co-regulation of T2D parameters and liver fibrosis risk. High NAFLD fibrosis scores (NFS) before intervention were associated with elevated HbA1c levels and T2D. Loss of weight and body fat percentage (BFL) were associated with improved glucose and lipid metabolism and reduced risk of NAFLD-related fibrosis, with particularly beneficial effects by RYGB. Both T2D improvement and NFS decrease were positively associated with high BFL. A highly significant correlation of NFS reduction with BFL was restricted to male patients while being absent in females, accompanied by generally higher BFL in men. Overall, the data display the relation of BFL, T2D improvement, and reduced NAFLD-related fibrosis risk during weight loss in morbidly obese individuals induced by diet or RYGB. Furthermore, our data suggest a considerable sexual dimorphism concerning the correlation of fat loss and improved risk of liver fibrosis.

## 1. Introduction

The metabolic syndrome (MetS) represents a severe health issue of global relevance [1] and comprises a number of morbidities, including obesity, hypertension, dyslipidemia, and type 2 diabetes mellitus (T2D) [2]. Importantly, metabolic dysregulation is closely related to concomitant inflammatory processes commonly summarized as “metaflammation” [3]. Non-alcoholic fatty liver disease (NAFLD) [4] is an important representative of metabolic morbidities [5,6] frequently accompanied by obesity and T2D [7]. As a major entity of chronic liver disease [7], NAFLD is, due to severe liver damage and hepatic malfunction by non-alcoholic hepatic steatosis [8], associated with considerably increased mortality depending on the severity of liver fibrosis [9,10,11]. Thus, NAFLD fibrosis score (NFS)—being calculated from the parameters albumin, age, aspartate transaminase/alanine aminotransferase (AST/ALT) ratio, BMI, hyperglycemia, and platelet count [8]—represents, among others, an important and non-invasive marker in order to indicate the risk of hepatic fibrosis [12]. Most important, genetically driven NAFLD promotes T2D and central obesity and vice versa, as was recently shown in a large-scale genome-wide association study (GWAS) approach [13]. Furthermore, optimal NFS cutoff values were reported to depend on the severity of obesity in order to rule out advanced hepatic fibrosis [11].

Recently, it was observed by liver biopsy that steatohepatitis represents the sole feature of liver damage in T2D. Almost all patients with T2D or MetS have NAFLD, which in patients with T2D means NASH [14].

The strict relationship between NAFLD and T2D involves some pathophysiological mechanisms still poorly studied or neglected today. In particular, the role of the opioid system, both on NAFLD [15] and on insulin resistance/obesity, needs further investigation [16]. Both physiological and pharmacological plasma levels of beta-endorphin are able to provoke marked islet hormone release in the early phase of human obesity [16]. In addition, we showed that the loss of endocannabinoid receptor 1 signaling led to reduced PLIN2 abundance, which triggers lipophagy [17].

Given the known causal relations of metabolic disorders [13], regulation and improvement of T2D and NAFLD under the terms of excessive weight loss in obese individuals represent a crucial issue for a better understanding of the interactions between distinct MetS comorbidities and for the development of therapeutic approaches applicable in order to improve obesity as well as glucose metabolism and liver integrity. Gastric sleeve and RYGB have been reported to significantly improve hepatic insulin sensitivity [18] and liver function in obesity-related NAFLD [19] and represent potential therapeutic strategies for NAFLD treatment [20,21]. In particular, a comparison of metabolic effects induced by bariatric surgery and diet as alternative approaches is of high interest [22].

The present study aimed to investigate and compare metabolic improvements regarding both hepatic injury and T2D in relation to weight loss induced by either invasive (RYGB) or conservative therapy (low-calorie formula diet) in a large and well-characterized cohort of morbidly obese individuals. In particular, the focus of the current study lies on the analysis of the association and possible interaction of T2D improvement and NAFLD attenuation during obesity therapy.

## 2. Materials and Methods

### 2.1. ROBS (Research in Obesity and Bariatric Surgery) Study Cohort

Serum samples were collected from the ROBS (Research in Obesity and Bariatric Surgery) study cohort [23]. ROBS is an open-label, non-randomized, monocentric, prospective and observational (explorative and confirmatory) study of patients routinely undergoing either bariatric surgery (gastric sleeve or Roux-en-Y gastric bypass) or a low-calorie formula diet (LCD) in the tertiary care centre at the University hospital of Giessen, Germany. The detailed information about this study cohort can be drawn from a previous publication [23]. The present study comprises data for ROBS subjects who completed visits V (base-line), V3, V6, and V12 (3, 6, and 12 months follow-up) and, thus, represents an extension of the study cohort introduced by Brock et al. in 2019 [23].

Briefly, patients were treated by a multidisciplinary team of physicians and professionals from Internal Medicine, Endocrinology/Diabetology, Metabolic/Visceral Surgery, Psychosomatic Medicine/Psychotherapy, Nutritional Science/Dietetics, and Sports Medicine at the Obesity Centre at the University of Giessen, Germany. The study was approved by the local ethical committee at the University of Giessen, Germany (file: AZ 101/14). All patients gave informed consent and were informed about the aim of the study. Data anonymization and privacy policy were accurately applied. Obese patients with a BMI > 40 kg/m^2^ or with a BMI > 35 kg/m^2^ and coexisting T2D were consecutively admitted for bariatric surgery from January 2015 to April 2021. Exclusion criteria were: pregnancy, evidence of or suspicion on underlying endocrine diseases, untreated bulimia nervosa and binge eating behaviour, use of illicit drugs, neoplasm, severe psychiatric disorders, psychosis, and psychopathologic instability.

In the present study, 160 obese patients were enrolled who either received a Roux-en-Y gastric bypass (RYGB, *n* = 79) or underwent conservative obesity therapy with low-calorie formula diet (LCD, *n* = 81). Liver fibrosis scores (BARD, NFS, FIB-4) and Albumin-Bilirubin (ALBI) score (as a predictor of hepatocellular carcinoma) were assessed according to current guidelines and applying established calculation formula [24,25,26,27].

#### 2.1.1. Roux-en-Y Gastric Bypass

In total, 79 patients receiving RYGB were included in the present study. Before admission for bariatric surgery, each patient underwent the following examinations (cited as base-line routine screening program): history and physical examination, 2 mg dexamethasone suppression test, routine clinical chemistry and endocrinology examination, 2 h oral glucose tolerance test (OGTT; non-diabetic patients only), esophagogastroduodenoscopy, abdominal ultrasound, long-term (24 h) blood pressure monitoring, 12-lead electrocardiography, screening for obstructive sleep apnoea, chest radiography, body plethysmography, bioimpedance analysis, echocardiography, and nutritional and psychosomatic counselling.

A surgeon at a single tertiary care centre performed the RYGB procedure. In this case, gastric bypass combined with a fundectomy as well as a circular gastrojejunostomy took place. In this setting, an 8–10 cm pouch was created, and the lengths of the biliopancreatic and alimentary limbs were set at 70–90 and 140–160 cm, respectively. A multidisciplinary group consisting of physicians and specialists in internal medicine, endocrinology/diabetology, metabolic/visceral surgery, psychosomatic medicine and psychotherapy, and nutritional science/dietetics and sports medicine from the University of Giessen provided further treatment.

#### 2.1.2. Low-Calorie Formula Diet

In total, 81 patients in the LCD group participated in a diet program starting with a 12-week fasting phase with five servings of food substitutes daily. This was followed by an 8-week conversion phase, during which portions are partially replaced by a mixed diet and, consecutively, by a stability phase without food substitutes being served. The diet program was accompanied by weekly group meetings that included medical check-up, exercise program, behavioural training, and a therapeutically guided group session.

### 2.2. Data Collection

Data collection was performed at four different time points, before RYGB surgery or the beginning of dietary intervention (V0) and after 3, 6, and 12 months (V3, V6, and V12). The examination of the patients included an anthropometric assessment and the collection of clinical and psychological data as well as medication, smoking habits, and nutritional status. In addition, a routine laboratory examination (CRLE) was performed and serum samples (21.5 mL) were collected in the fasting state for subsequent quantifications. Complete data on the assessed parameters are available for all study subjects with few exceptions. The individual parameters measured are shown in Table 1, Table 2 and Table 3.

### 2.3. Statistical Analysis

For explorative data analysis, a statistical software package (SPSS 26.0) was used. Non-parametric numerical parameters were analyzed by the Mann–Whitney U-test (for 2 unrelated samples), the Kruskal–Wallis test (>2 unrelated samples), the *Wilcoxon* test (for 2 related samples) or the Friedman test (>2 related samples). Correlation analysis of parameters was performed applying non-parametric Spearman-rho test. Distribution and relationship of categorial variables were analyzed applying chi-square statistics (for unrelated samples) and McNamere test (for related samples). *p* values below 0.05 (two tailed) was considered as statistically significant. In the figures, means are displayed as dots with whiskers giving the standard error of the mean (1 × SEM). Box plots are indicating median, upper/lower quartiles, interquartile range, minimum/maximum values and outliers.

## 3. Results

### 3.1. Characteristics of the Study Cohort

A total of 81 obese patients enrolled on a low-calorie formula diet program were included in the present study. Characteristics concerning anthropometric and physiological parameters are depicted by Table 1. Patients experienced a significant loss of body weight. Mean BMI was reduced by 10.1 kg/m^2^ (*p* < 0.001).

Among indices of liver function, distributions of ALBI and BARD were significantly changed towards elevated score levels after 12 months of LCD-induced weight loss, whereas NFS and FIB-4 remained unaltered (Table 1).

Characteristics of 79 patients undergoing bariatric surgery (RYGB) are given in Table 2. Compared to LCD participants, loss of body weight and BMI (−18.6 kg/m^2^) induced by RYGB was even more pronounced. Importantly and unlike the LCD group, the proportion of diabetic patients was significantly reduced at 12 months follow-up (*p* < 0.001) upon RYGB surgery.

Except for a slight elevation in ALBI scores, the distribution of liver indices was not significantly changed during 12 months following RYGB (Table 2). In contrast, serum ALT and GGT, both indicating acute hepatic injury, were reduced by LCD and RYGB.

There was a higher proportion of females among RYGB patients and they exhibited significantly higher mean body weight, body fat percentage, and BMI (see comparison of general characteristics at base-line and at 12 months follow-up in Table 3). Of note, the latter differences were abolished by more efficient body weight loss and excessive weight loss during 12 months in the RYGB sub-cohort.

Overall, *n* = 117 female patients participated in the overall study cohort with a mean age of 41.4 ± 11.5 years at study base-line. Among these, 28 women (23.9%) had a base-line age of over 50 years, which might be considered a post-menopausal age.

### 3.2. Base-Line NAFLD Fibrosis Scores Are Associated with T2D

**Figure 1:**
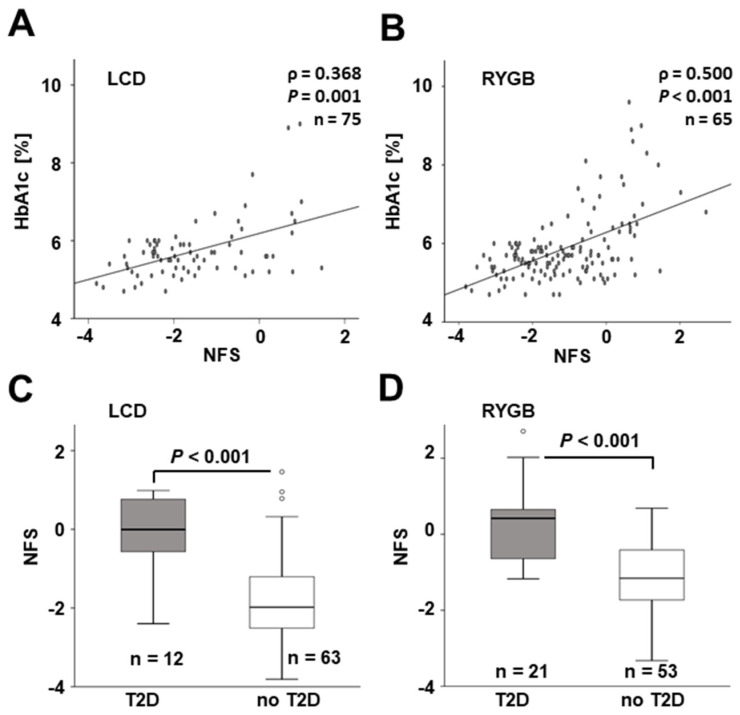
**Association of base-line NAFLD fibrosis score with T2D.** Base-line HbA1c levels and NFS were positively correlated (**A**,**B**) and mean NFS was significantly elevated in T2D patients (**C**,**D**) in both sub-cohorts of the study. HbA1c, glycosylated hemoglobin; LCD, low-calorie formula diet; NFS, NAFLD fibrosis score; RYGB, Roux-en-Y gastric bypass; T2D, type 2 diabetes mellitus.

Both in LCD (Figure 1A) and RYGB patients (Figure 1B), NAFLD fibrosis scores were significantly correlated with HbA1c levels before the beginning of intervention (*p* = 0.001 and *p* < 0.001, respectively). Accordingly, NFS values were significantly elevated in patients suffering from T2D when compared to individuals without T2D in both subgroups of the study cohort (Figure 1C,D).

### 3.3. Dynamics of Obesity-Related Parameters and Serum Lipids during Therapy-Induced Weight Loss

**Figure 2:**
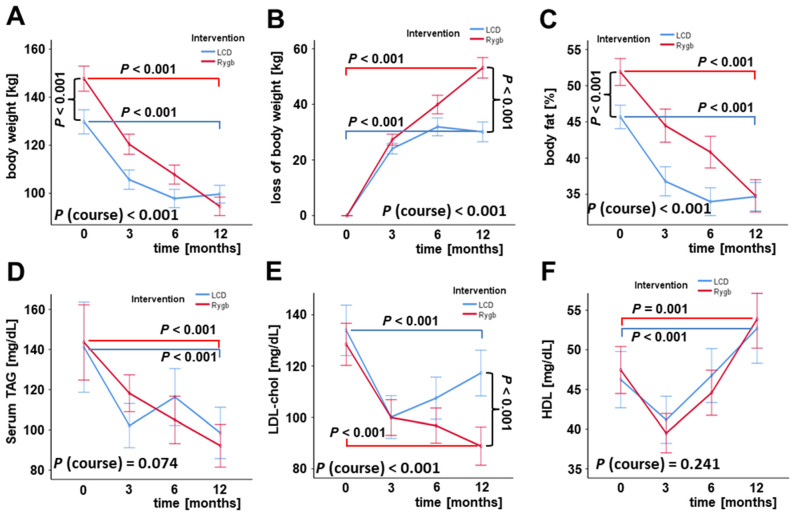
**Improvement of metabolic parameters during 12 months of weight loss in both LCD and RYGB patients.** For LCD and RYGB patients, body weight (**A**), body weight loss (**B**), body fat percentage (**C**), serum TAG (**D**) and serum cholesterol levels (**E**,**F**) are displayed at base-line and at 3, 6, and 12 months after the beginning of intervention. HDL, high-density lipoprotein particle cholesterol; LCD, low-calorie formula diet; LDL, low-density lipoprotein particle cholesterol; RYGB, Roux-en-Y gastric bypass; TAG, triglycerides.

As is illustrated in Figure 2, RYGB and LCD both induced a significant and consistent loss of body weight and body fat percentage (Figure 2A–C), with RYGB patients starting from significantly higher levels at base-line, and an improved systemic lipid profile (Figure 2D–F) during 12 months post-surgery or after the start of the diet program, respectively. The favorable effects on body weight, fat percentage, and LDL levels were more pronounced in bariatric surgery patients (Figure 2A,B,E).

### 3.4. Improvement of T2D and NAFLD Occurs during Weight Loss

**Figure 3:**
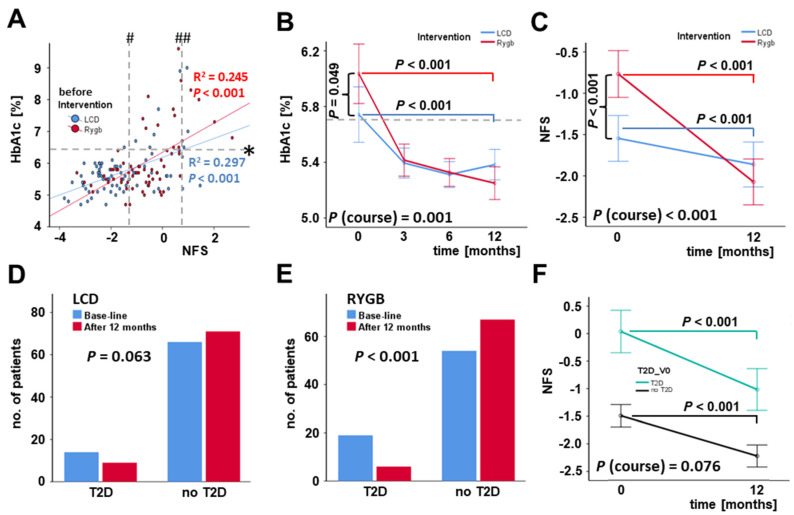
Changes in HbA1c levels, NAFLD fibrosis score, and T2D prevalence during weight loss induced by bariatric surgery (RYGB) or low-calorie formula diet (LCD). Blood HbA1c levels and NFS were positively correlated at base-line levels (**A**) and were significantly improved under weight loss (**B**,**C**). Considerable T2D improvement was associated with weight loss after RYGB but not during LCD (**D**,**E**). NFS was significantly reduced during 12 months in LCD and RYGB sub-cohorts (**F**). HbA1c, glycosylated hemoglobin; NFS, NAFLD fibrosis score; T2D, type 2 diabetes mellitus. Lower and higher cutoff NFS and HbA1c (in obesity): # NFS < −1.455 no fibrosis ## NFS > 0.76 fibrosis likely * HbA1c ≥ 6.5 diabetes.

Body weight loss, body fat loss, and improved systemic lipid profile upon intervention were accompanied by further beneficial metabolic effects. Logistic regression analysis confirmed a significant relation of blood HbA1c levels—as an important and long-term marker of hyperglycemia and T2D—and NFS for the overall study cohort (Figure 3A). HbA1c levels were significantly decreased 12 months after RYGB surgery or the beginning of dietary intervention, respectively (Figure 3B). Of note, this improvement of hyperglycemia was accompanied by lowered mean NAFLD fibrosis scores (NFS) after weight loss (Figure 3C), with a particularly strong impact of RYGB. Statistical analysis of base-line and 12 months follow-up distribution concerning individuals with or without manifested T2D revealed no significant differences for the cohort of LCD participants (Figure 3D), whereas a significant improvement of T2D (defined as HbA1c below 6.5% at 12 months follow-up without insulin, GLP1 analogs, and oral anti-diabetic medication) was observed among bariatric surgery patients after RYGB (Figure 3E). Study subjects, both with or without T2D, exhibited a significant decrease in NFS during therapy-induced weight loss (Figure 3F).

**Figure 4:**
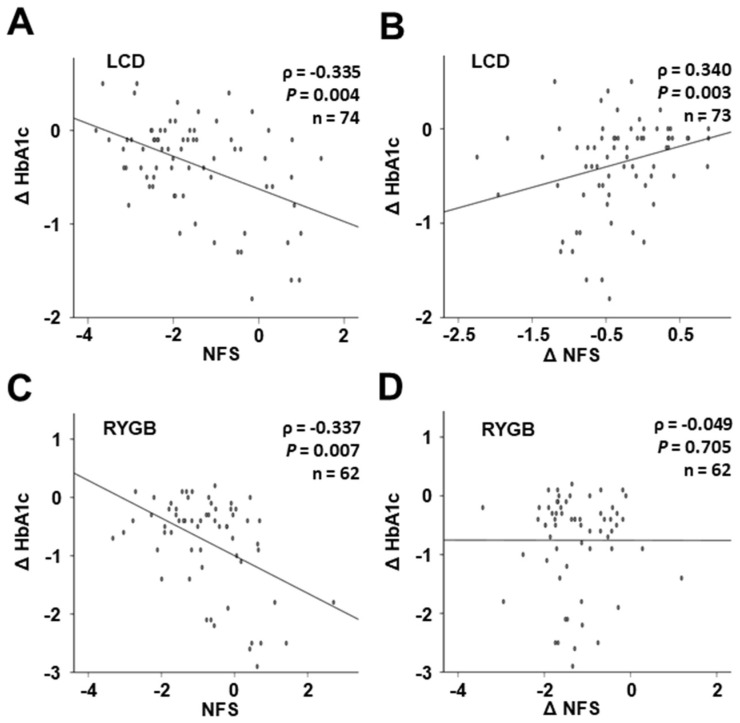
Correlation of base-line NFS and Δ NFS with Δ HbA1c during weight loss under low-calorie formula diet (**A**,**B**) and following RYGB (**C**,**D**). HbA1c, glycosylated hemoglobin; LCD, low-calorie formula diet; NFS, NAFLD fibrosis score; RYGB, Roux-en-Y gastric bypass; T2D, type 2 diabetes mellitus.

Of note, changes in HbA1c levels during weight loss were strongly correlated with base-line NFS both in the LCD and the RYGB sub-cohort (Figure 4A,C). Furthermore, Δ HbA1c was positively correlated with Δ NFS during 12 months of LCD-induced weight loss (Figure 4B), whereas this correlation was absent among RYGB patients (Figure 4D).

### 3.5. Relation of T2D Improvement to Body Fat Loss and Changes in NAFLD Fibrosis Score

**Figure 5:**
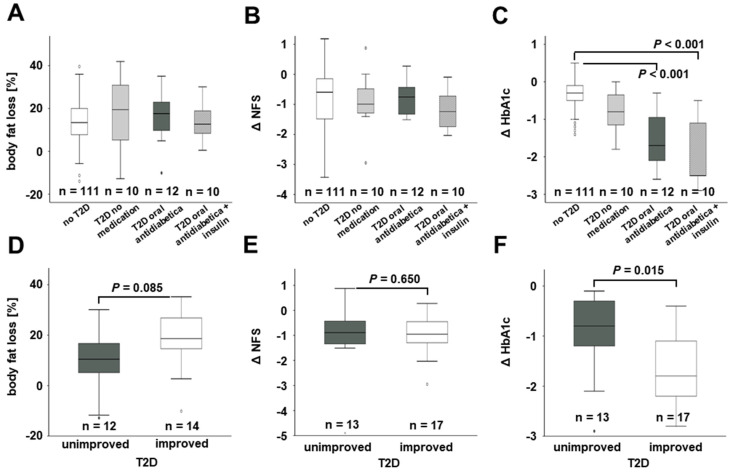
**Relation of body fat loss, HbA1c levels, and NFS to T2D improvement during 12 months in the overall study cohort.** Loss of body fat percentage (**A**) and Δ NFS (**B**) during 12 months were independent of T2D presence and medication. Decrease in HbA1c levels during weight loss was pronounced in T2D patients receiving medication (**C**). Body fat loss, Δ NFS, and Δ HbA1c were not significantly associated with T2D improvement (**D**–**F**). HbA1c, glycated hemoglobin; NFS, NAFLD fibrosis score; T2D, type 2 diabetes mellitus.

As depicted in Figure 5, both body fat loss (Figure 5A) and improvement of NFS (Figure 5B) was not different between non-diabetic individuals and diabetic patients independent of any diabetes medication. The observed dietary-induced and post-surgery decline of blood HbA1c levels was more pronounced in diabetic patients receiving insulin therapy and/or oral antidiabetics when compared to non-diabetic and untreated diabetic individuals (Figure 5C).

There was a non-significant trend towards a higher mean body fat percentage loss in diabetic patients experiencing T2D improvement (defined as HbA1c below 6.5% at 12-month follow-up without insulin, GLP-1 analogs or oral anti-diabetic medication) during 12 months of weight loss (*p* = 0.085; Figure 5D), whereas NFS changes were not different for unimproved and improved T2D individuals (Figure 5E). HbA1c levels were more strongly decreased in patients with T2D improvement (*p* = 0.015; Figure 5F).

### 3.6. NAFLD Fibrosis Score Improved More in Patients with High Body Fat Loss

**Figure 6:**
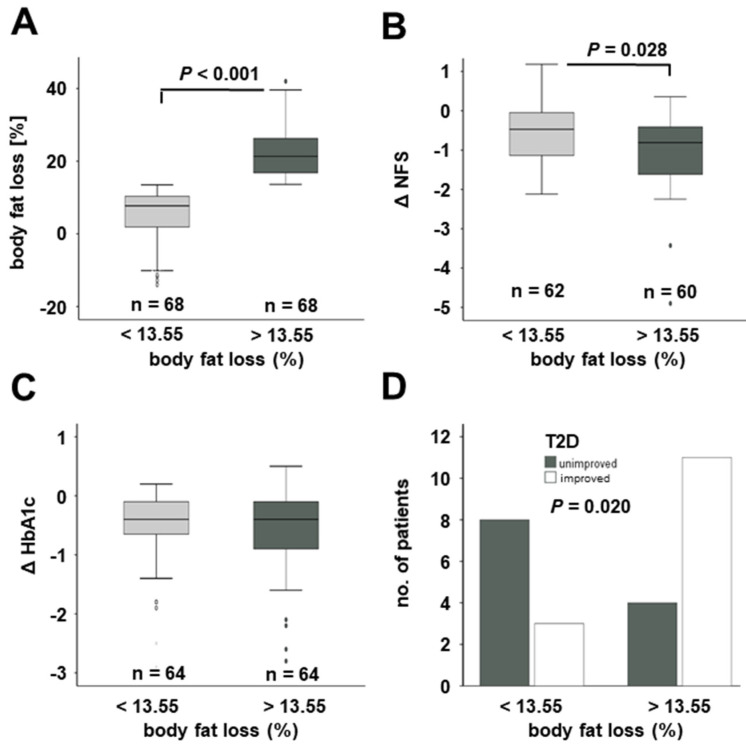
**NAFLD improvement and T2D improvement depend on the extent of body fat loss within the whole study cohort.** Patients with above-median body fat loss (**A**) exhibited significantly stronger reduction in NFS (**B**). Changes in HbA1c levels were equal in patients below and above median body fat loss (**C**). High body fat loss was associated with significantly higher proportion of T2D improvement among diabetic patients (*n* = 26) (below median: 3 out of 11 T2D patients improved; above median: 11 out of 15) (**D**). HbA1c, glycosylated hemoglobin; T2D, type 2 diabetes mellitus.

For subgroup analysis, the study cohort was subdivided into weak and strong responders towards obesity therapy concerning change of body fat percentage (Figure 6). Within the whole study cohort, weak responders (*n* = 68) experienced an average loss of 5.47% of body fat compared to a significantly higher loss of 22.4% in the subgroup of strong responders (*n* = 68) (Figure 6A). As indicated in Figure 6B, strong responders also exhibited a stronger reduction in NAFLD fibrosis score (*p* = 0.028), whereas the decrease in HbA1c levels did not differ between both subgroups (Figure 6C). However, when focusing on patients with diagnosed T2D at study base-line (*n* = 26), there was a significantly higher proportion of T2D improvement among “strong responders” than among “weak responders” (Figure 6D).

### 3.7. Correlation of Body Fat Loss and NAFLD Improvement Is Pronounced in Males

**Figure 7:**
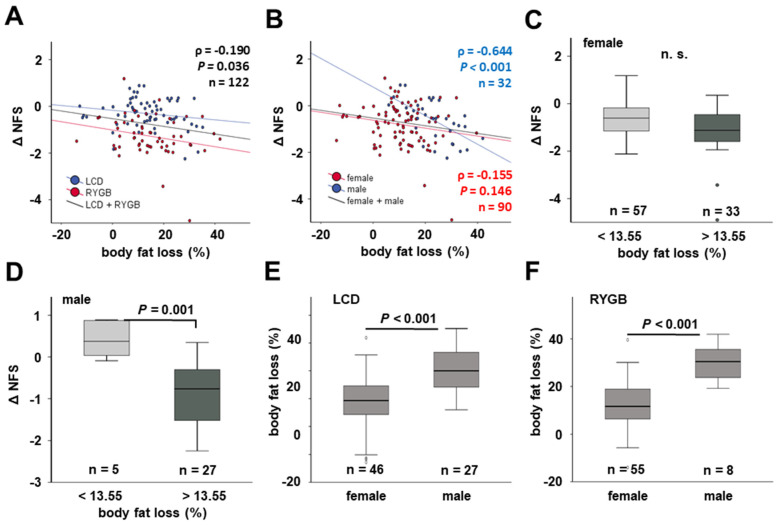
**The correlation of NFS change with body fat loss after 12 months depends on sex.** NFS changes during therapeutic intervention were negatively correlated with body fat loss (**A**), which was particularly pronounced in male patients (**B**). Unlike women (**C**), men exhibited a strong association of body fat loss and ΔNFS (**D**). Loss of body fat percentage was higher in male than in female patients in both sub-cohorts of the study (**E**,**F**). LCD, low-calorie formula diet; NFS, NAFLD fibrosis score, n.s., not significant; RYGB, Roux-en-Y gastric bypass.

The extent of body fat loss was negatively correlated with changes in NAFLD fibrosis score (rho = −0.190, *p* = 0.036), i.e., was correlated with NFS reduction (Figure 7A), without differences between the subgroups of conservatively treated and bariatric patients. Of note, a sexual dimorphism was observed. While there was a trend among female patients (rho = −0.155, *p* = 0.146), the negative correlation was considerably strong among men (rho = −0.644, *p* < 0.001) (Figure 7B).

Further stratified subgroup analysis revealed that the significant association of “high” body fat loss (>13.55%) to NFS change displayed in Figure 6B was absent in female patients (Figure 7C), while being highly significant among male patients (Figure 7D). This observed sexual divergence proved to be consistent for women at both age subgroups (≤ and >50 years at base-line), designating the beginning of post-menopausal age (data not shown). Furthermore, 12 months’ worth of loss of body fat percentage was significantly stronger in men than in women under LCD (Figure 7E) as well as following RYGB surgery (Figure 7F).

## 4. Discussion

The present study investigates NAFLD and T2D prevalence within a large obesity cohort, as well as the impact of both conservative and bariatric (Roux-en-Y gastric bypass, RYGB) obesity therapy, on the improvement of these metabolic morbidities. Although the two sub-cohorts of patients differed significantly in the severity of metabolic disorders—such as BMI, excessive weight, body fat percentage, and HbA1c levels—associated with obesity, a general correlation of NAFLD fibrosis score (NFS) with T2D was observed in both cohorts.

During 12 months of therapy-induced weight loss, a significant improvement in the systemic parameters of both lipid and glucose metabolism occurred in LCD and RYGB patients in accordance with previous studies [18,28]. Importantly, RYGB turned out to be more effective both in terms of weight loss and rate of T2D improvement, resulting in a significantly reduced proportion of diabetic individuals in the bariatric sub-cohort 12 months after surgery. When interpreting the pronounced weight loss following bariatric surgery, it is important to consider the significantly higher mean body weight and body fat percentage of these patients when compared to those in the LCD group at base-line.

NFS was significantly reduced by LCD as well as by RYGB, which is in accordance with beneficial effects on NAFLD having been reported for bariatric surgery [19,29]. Appearing somewhat contradictory, mean BARD scores addressing liver fibrosis increased during 12 months of weight loss in LCD patients. However, the strong impact of an elevated AST/ALT ratio on BARD score has to be considered, which is not specific for hepatic damage. In a recently published study, the BARD score displayed a lack of specificity for hepatic fibrosis compared to FIB-4, which is in line with our present results [30]. Furthermore, BARD is primarily applicable in order to predict manifested and advanced fibrosis in NAFLD, whereas NFS represents a reliable tool for the exclusion of individuals with a low risk of fibrosis [31]. The latter, therefore, was selected for evaluation of the present study cohort consisting of patients without diagnosed liver fibrosis.

Overall, both patients with manifest T2D and patients without T2D in the whole study cohort experienced a significant reduction in NFS during 12 months of intervention. Whilst significant differences concerning body fat loss or changes in NFS were observed between diabetic and non-diabetic patients, changes of HbA1c levels during weight loss—indicating improved glucose metabolism—were negatively correlated with base-line NFS. Similarly, Vangoitsenhoven et al. recently reported a positive association of liver steatosis with RYGB-induced T2D remission [32]. Thus, the present data suggest that patients with an increased risk of liver fibrosis might take particular advantage of both LCD and RYGB in terms of diabetes improvement and carbohydrate metabolism. We observed that changes of NFS and HbA1c levels were positively correlated within the LCD sub-cohort but not among bariatric surgery patients, indicating putative interrelations between weight-loss-dependent processes improving liver function and glucose metabolism that appear to be exclusive for dietary intervention. This finding is particularly interesting since, to the best of our knowledge, the present study represents the first examination of the relation between NAFLD fibrosis risk and HbA1c dynamics directly comparing effects of bariatric surgery and the here-applied setting of a balanced low-calorie formula diet in a large cohort of obese individuals [23]. Of note, as was recently summarized by Watanabe et al. [33], diet regimens including strong caloric restriction, especially some ketogenic diets, exert beneficial effects on NAFLD. Since LCD in the present study is not ketogenic, the observed effects are likely to be mediated not by physiological mechanisms involving forced synthesis of ketone bodies but rather by hypocaloric conditions per se. A Taiwanese study investigating the effects of two different very-low-calorie diets (12 weeks; 450 and 800 kcal/day, respectively) observed improvement of NAFLD as well as blood glucose levels [34]. Similarly, Schwenger et al. reported improved NAFLD and lowered HbA1c levels in morbidly obese individuals after attending a pre-bariatric very-low-calorie diet [35]. It, therefore, appears reasonable to assume that the correlation of NFS and HbA1c decline in the present LCD sub-cohort is mainly due to parallel hypocaloric effects on both parameters.

Metabolic alterations are caused by several factors. For example, hepatitis C virus (HCV) infection is now considered to cause metabolic alterations instead of simply being a viral infection. Recently, a prospective multicenter case-control study showed that HCV clearance by direct-acting antiviral (DAA) treatment reduces T2D incidence probably by restoring the HCV-induced alteration of glucose homeostasis mechanisms [36].

A stronger decrease in NFS was detected in patients who experienced higher body fat loss, i.e., higher than the median of 13.55% (“strong responders”) compared to patients with a lower body fat loss. Regarding the change in body fat percentage as a valid indicator of obesity therapy success, it, therefore, seems reasonable to conclude that a reduced risk of NAFLD-related fibrosis is correlated with successful fat loss. Furthermore, over-median body fat percentage loss was accompanied by a significantly higher proportion of T2D improvement among diabetic individuals when compared to the group of patients with less pronounced reduction in relative fat mass.

NAFLD and IR are bidirectionally correlated and, consequently, the development of pre-diabetes and diabetes is the most direct consequence at the extrahepatic level. A very recent review explains in an updated and complete way the pathophysiological mechanisms that support this relationship [37]. Current NAFLD guidelines were designed to be relevant to practice and show a clear way out of the current drug-based “therapeutic nihilism”. Diagnostic and therapeutic algorithms are based on metabolic comorbidities, e.g., T2D and fibrosis stage to improve applicability [38].

For the whole study cohort, an only modest negative correlation of NFS change and body fat loss was observed. Most interestingly, subgroup analysis revealed a very strong negative correlation of these parameters among male patients. Absence of this negative correlation in females indicates a potential sexual dimorphism concerning the association of body fat loss and reduced NAFLD-related fibrosis risk. Compared to men, women are better protected against NAFLD [39] and visceral obesity [40] in premenopausal age. Rather complex sex differences are known in the context of dysregulated glucose metabolism, with higher prevalence of impaired fasting glucose in men and rather prevalent impairment of glucose intolerance in women [41]. Given the positive correlation of NAFLD risk with visceral fat accumulation [13], which is predominantly abundant in male obesity [40], the observed strong correlation of body fat loss and NFS decrease might be explained by the reduction in visceral rather than subcutaneous fat mass in men. In the present study, male patients overall experienced a significantly stronger loss of body fat percentage than females both in the LCD and RYGB group. This is in accordance with previous reports on a sexual dimorphism in long-term laparoscopic surgery effects with higher weight loss in male patients [42]. Therefore, it appears reasonable to assume that the pronounced reduction in body fat percentage in men might be causally linked with a stronger decrease in NAFLD-related fibrosis risk. On the other hand, a recent study from Schmitz et al. suggested male sex as one of several factors impeding the recovery of liver function in bariatric surgery [19]. Thus, a rather complex sexual dimorphism regarding the therapeutic impact of diet and bariatric surgery both on obesity and liver fibrosis should definitely be considered and will have to be examined in detail by further studies. Future approaches addressing this issue should investigate effects of predominantly male/female fat distribution as well as sexual hormones on mechanisms of fat loss and associated improvements concerning metabolism and metaflammation. Of particular interest, estrogens have a crucial and beneficial role in liver function, such as glucose and lipid homeostasis, as well as in NAFLD and NASH [43]. Post-menopausal women exhibit an increased risk of severe liver fibrosis in NASH, which can be significantly attenuated by estrogen replacement therapy [44]. On the other hand, androgens appear to exhibit a rather ambiguous relationship to fatty liver disease phenotype, e.g., while dihydrotestosterone was reported to exert protective effects against NAFLD in a male rat model [45], testosterone might represent a risk factor for NASH and associated fibrosis in young women [46]. In general, published data on sexual hormones affecting the relation of NAFLD fibrosis and therapeutically induced fat loss in obese individuals under therapeutic conditions comparable to the present study are scarce. Since this issue was not in the primary focus of the ROBS study and relevant sexual hormones, therefore, have not been quantified throughout the time-course of weight loss, their potential role in the observed differences between female and male patients remains somewhat speculative at the present state. Of note, we did not observe an impact of female age on this sexual divergence, thus rather questioning a potential role of post-menopausal age and associated changes in estrogen levels. Furthermore, a previous study reported elevated NAFLD risk in menopausal and post-menopausal women not to be correlated with estrogen replacement [47], arguing for a rather complex hypothetical relation between female sexual hormones and fatty liver diseases. This issue and its implicit potential for innovative therapy options will have to be specifically addressed by future clinical investigation and evaluation.

## 5. Conclusions

Non-alcoholic fatty liver disease (NAFLD)-related fibrosis score (NFS) in obesity is associated with type 2 diabetes mellitus (T2D) prevalence and is positively correlated with plasma HbA1c levels. During therapy-induced weight loss, both T2D improvement and the decrease in NFS are correlated with loss of body fat percentage whilst not being significantly correlated with each other. Pronounced loss of body fat is associated with improved fibrosis risk in men but not in women. In summary, our data suggest that the improvement of T2D and the improvement of NAFLD (especially fibrosis) are associated with the success of obesity therapy.

## Figures and Tables

**Table 1:** Base-line and 12 months follow-up characteristics of obese patients attending the low-calorie formula diet program (*n* = 81). Means are depicted and ranges of values are given in brackets.

Parameters	Base-Line	12 Months Follow-Up	*p*
**A** **Anthropometric Characteristics**			
**Demographic**			
Age [years]	42.8 (20; 67)	-	-
Gender			-
Female	52 (64.2%)	-	
Male	29 (35.8%)	-	
**Anthropometric**			
BMI [kg/m^2^]	43.6 (31.9; 59.2)	33.5 (24.3; 49.7)	<0.001
Body weight [kg]	130 (90.1; 185.4)	99.6 (61; 159)	<0.001
Weight loss [%]	-	23 (1; 41.4)	-
Excessive weight [kg]	61.3	-	-
Excessive weight loss [%]	-	50.4	-
Body fat [%]	45.9 (28.5; 59.2)	34.8 (15.0; 53.7)	<0.001
Waist–hip ratio	0.95 (0.69; 1.25)	0.9 (0.72; 1.13)	<0.001
**B** **Anamnesis and medication**			
Hypertension			<0.001
Yes	40 (49.4)	26 (32.1 %)	
no	40 (49.4)	54 (66.7)	
Cardiovascular disease			0.317
Yes	2 (2.5)	3 (3.7)	
No	79 (97.5)	77 (95.1)	
Smoking			0.564
Yes	18 (22.2)	19 (23.5)	
No	63 (77.8)	61 (75.3)	
Hormonal contraception			0.059
Yes	12 (14.8)	16 (19.8)	
No	67 (82.7)	64 (79)	
**C** **Metabolism**			
Type 2 diabetes mellitus			0.063
Yes	14 (17.3)	9 (11.1)	
No	66 (81.5)	71 (87.7)	
Hyperlipidemia			0.001
Yes	37 (45.7)	21 (25.9)	
No	44 (54.3)	59 (72.8)	
Number of medications			0.041
0	72 (87.8)	79 (96.3)	
1	6 (7.3)	0	
2	2 (2.4)	1 (1.2)	
3	1 (1.2)	0	
Insulin therapy			0.157
Yes	5 (6.1)	3 (3.7)	
No	76 (93.9)	77 (93.9)	
GLP Analogs			0.157
Yes	2 (2.5)	0	
No	79 (97.5)	80 (98.8)	
LDL cholesterol [mg/dL]	132.7 (40; 201)	114.5 (44; 213)	<0.001
HDL cholesterol [mg/dL]	48.4 (29; 84)	51.5 (28; 77)	0.001
Total cholesterol [mg/dL]	193.8 (135; 260)	177.8 (94; 299)	<0.001
Serum triglycerides [mg/dL]	138 (48; 436)	99 (39; 283)	<0.001
CRP [mg/L]	9.2 (0.6; 31.0)	5,1 (0.5; 152)	<0.001
HbA1c [%]	5.7 (4.7; 9.0)	5.4 (4.5; 7.7)	<0.001
**Liver**			
ALT [U/L]	36.5 (11; 132)	29.8 (8; 418)	<0.001
AST [U/L]	25.1 (8; 54)	24.5 (11; 312)	0.008
Alkaline phosphatase [U/L]	75.8 (35; 122)	68.8 (30; 194)	<0.001
GGT [U/L]	33.3 (9; 136)	28.5 (5; 514)	<0.001
Bilirubin [µmol/L]	10.3 (3.4; 24)	12.4 (3.4; 44.5)	0.002
Albumin [g/L]	44.2 (36.8; 50.2)	43,6 (38.3; 52.8)	0.060
**D** **Liver scores**			
**BARD**			0.009
0	0	3 (3.7)	
1	38 (46.9)	23 (28.4)	
2	10 (12.3)	11 (13.6)	
3	30 (37)	37 (45.7)	
4	3 (3.7)	7 (8.6)	
**ALBI**			<0.001
1	11 (13.6)	6 (7.4)	
2	64 (79)	71 (87.7)	
3	0	1 (1.2)	
**NFS**			1.000
<−1.445	46 (56.8)	48 (59.3)	
>0.675	8 (9.9)	3 (3.7)	
**FIB-4**			1.000
<1.45	70 (86.4)	67 (82.7)	
>3.25	0	1 (1.2)	

**Table 2:** Base-line and 12 months follow-up characteristics of bariatric surgery patients receiving Roux-en-Y gastric bypass (*n* = 79). Means are depicted and ranges of values are given in brackets.

	Base-Line	12 Months Follow-Up	*p*
**A** **Anthropometric Characteristics**			
**Demographic**			
Age [years]	40.7 (20; 60)	-	-
Gender			-
Female	65 (82.3%)	-	
Male	14 (17.7%)	-	
**Anthropometric**			
BMI [kg/m^2^]	51.7 (42; 62)	33.1 (24; 42)	<0.001
Body weight [kg]	149.4 (109; 244)	94.6 (61; 146)	<0.001
Weight loss [%]		35.45 (16.75; 54.91)	-
Excessive weight [kg]	82.9	-	-
Excessive weight loss [%]		64.4	-
Body fat [%]	52 (30; 62.1)	35.5 (19.6; 49.1)	<0.001
Waist–hip ratio	0.96 (0.71; 1.33)	0.88 (0.71; 1.05)	<0.001
**B** **Medical history and medication**			
Hypertension			<0.001
Yes	51 (64.6)	30 (38)	
no	26 (32.9)	45 (57)	
Cardiovascular disease			1.000
Yes	3 (3.8)	2 (2.5)	
No	74 (93.7)	74 (93.7)	
Smoking			0.655
Yes	22 (27.8)	21 (26.6)	
No	57 (72.2)	55 (69.6)	
Hormonal contraception			0.564
Yes	14 (17.7)	12 (15.2)	
No	63 (79.7)	64 (81)	
**C** **Metabolism**			
Diabetes mellitus type 2			<0.001
Yes	19 (24.1)	6 (7.6)	
No	54 (68.4)	67 (84.8)	
Hyperlipidemia			<0.001
Yes	32 (40.5)	9 (11.4)	
No	47 (59.5)	65 (82.3)	
Number of medications			0.001
0	60 (75.9)	71 (89.9)	
1	11 (13.9)	3 (3.8)	
2	6 (7.6)	1 (1.3)	
3	2 (2.5)	0	
Insulin therapy			0.005
Yes	11 (13.9)	0	
No	67 (84.8)	74 (93.7	
GLP Analogs			0.046
Yes	4 (5.1)	0	
No	75 (94.9)	75 (94.9)	
LDL cholesterol [mg/dL]	128.67 (53; 233)	88,13 (13; 153)	<0.001
HDL cholesterol [mg/dL]	47.60 (0; 87)	53.08 (17; 144)	<0.001
Total cholesterol [mg/dL]	185.5 (115; 290)	154 (96; 242)	<0.001
Serum triglycerides [mg/dL]	142.46 (58; 751)	90.19 (43; 253)	<0.001
CRP [mg/L]	14.3 (2.09; 110,89)	3.37 (0,49; 43,0)	<0.001
HbA1c [%]	6.0 (4.7; 9.6)	5.3 (4.4; 6.7)	<0.001
**Liver**			
ALT [U/L]	41.93 (12; 263)	29.92 (9; 186)	<0.001
AST [U/L]	29.16 (12; 140)	22.33 (8; 137)	<0.001
Alkaline phosphatase [U/L]	83.227 (36; 131)	82.71 (31; 270)	0.449
GGT [U/L]	64.99 (9; 1867)	20.69 (5; 279)	<0.001
Bilirubin [µmol/L]	8.80 (3.4; 23.9)	10.2396 (1.71; 25.66)	<0.001
Albumin [g/L]	43.5 (36.1; 51.0)	43.525 (37.4; 51.2)	0.980
**D** **Liver scores**			
**BARD**			0.093
0	0	3 (3.8)	
1	37 (46.8)	23 (29.1)	
2	15 (19)	14 (17.7)	
3	19 (24.1)	30 (38)	
4	8 (10.1)	9 (11.4)	
**ALBI**			0.037
1	15 (19)	11 (13.9)	
2	60 (75.9)	65 (82.3)	
3	0	0	
**NFS**			1.000
<−1.445	18 (22.8)	52 (65.8)	
>0.675	6 (7.6)	0	
**FIB-4**			1.000
<1.45	75 (94.9)	72 (91.1)	
>3.25	0	0	

**Table 3:** Comparison of base-line and 12 months follow-up characteristics of RYGB and LCD patients.

	Base-Line			12 months Follow-Up		
**A** **Anthropometric Characteristics**						
**Demographic**	**LCD** ***n*** **= 81**	**RYGB** ***n*** **= 79**	***p***	**LCD** ***n*** **= 81**	**RYGB** ***n*** **= 79**	***p***
Age [years]	42.8 (20; 67)	40.7 (20; 60)	0.282	-	-	
Gender						-
Female	52 (64.2%)	65 (82.3%)	0.01	-	-	
Male	29 (35.8%)	14 (17.7%)		-	-	
**Anthropometric**						-
BMI [kg/m^2^]	43.6 (31.9; 59.2)	51.7 (42; 62)	<0.001	33.5 (24.3; 49.7)	33.1 (24; 42)	0.755
Body weight [kg]	130 (90.1; 185.4)	149.4 (109; 244)	<0.001	99.6 (61;159)	94.6 (61; 146)	0.199
Weight loss [%]	-	-	-	23 (1; 41.4)	35.45 (16.75; 54.91)	<0.001
Excessive weight [kg]	61.3	82.9	<0.001	-	-	-
Excessive weight loss [%]	-		-	50.4	64.4	<0.001
Body fat [%]	45.9 (28.5; 59.2)	52 (30; 62.1)	<0.001	34.8 (15.0; 53.7)	35.5 (19.6; 49.1)	0.656
Waist–hip ratio	0.95 (0.69; 1.25)	0.96 (0.71; 1.33)	0.962	0.9 (0.72; 1.13)	0.88 (0.71; 1.05)	0.968
**B** **Medical history and medication**						
Hypertension			0.04			0.333
Yes	40 (49.4)	51 (64.6)		26 (32.1 %)	30 (38)	
no	40 (49.4)	26 (32.9)		54 (66.7)	45 (57)	
Cardiovascular disease			0.61			0.693
Yes	2 (2.5)	3 (3.8)		3 (3.7)	2 (2.5)	
No	79 (97.5)	74 (93.7)		77 (95.1)	74 (93.7)	
Smoking			0.413			0.580
Yes	18 (22.2)	22 (27.8)		19 (23.5)	21 (26.6)	
No	63 (77.8)	57 (72.2)		61 (75.3)	55 (69.6)	
Hormonal contraception			0.617			0.495
Yes	12 (14.8)	14 (17.7)		16 (19.8)	12 (15.2)	
No	67 (82.7)	63 (79.7)		64 (79)	64 (81)	
**C** **Metabolism**						
Diabetes mellitus			0.2			0.529
Yes	14 (17.3)	19 (24.1)		9 (11.1)	6 (7.6)	
No	66 (81.5)	54 (68.4)		71 (87.7)	67 (84.8)	
Hyperlipidemia			0.51			0.028
Yes	37 (45.7)	32 (40.5)		21 (25.9)	9 (11.4)	
No	44 (54.3)	47 (59.5)		59 (72.8)	65 (82.3)	
Number of medications			0.03			0.157
0	72 (87.8)	60 (75.9)		79 (96.3)	71 (89.9)	
1	6 (7.3)	11 (13.9)		0	3 (3.8)	
2	2 (2.4)	6 (7.6)		1 (1.2)	1 (1.3)	
3	1 (1.2)	2 (2.5)		0	0	
Insulin therapy			0.098			0.094
Yes	5 (6.1)	11 (13.9)		3 (3.7)	0	
No	76 (93.9)	67 (84.8)		77 (93.9)	74 (93.7	
GLP Analogs			0.389			1.000
Yes	2 (2.5)	4 (5.1)		0	0	
No	79 (97.5)	75 (94.9)		80 (98.8)	75 (94.9)	
LDL cholesterol [mg/dL]	132.7 (40; 201)	128.67 (53; 233)	0.384	114.5 (44; 213)	88,13 (13; 153)	<0.001
HDL cholesterol [mg/dL]	48.4 (29; 84)	47.60 (0; 87)	0.877	51.5 (28; 77)	53.08 (17; 144)	0.823
Total cholesterol [mg/dL]	193.8 (135; 260)	185.5 (115; 290)	0.102	177.8 (94; 299)	154 (96; 242)	<0.001
Serum triglycerides [mg/dL]	138 (48; 436)	142.46 (58; 751)	0.917	99 (39; 283)	90.19 (43; 253)	0.212
CRP [mg/L]	9.2 (0.6; 31.0)	14.3 (2.09; 110,89)	<0.001	5,1 (0.5; 152)	3.37 (0,49; 43,0)	0.060
HbA1c [%]	5.7 (4.7; 9.0)	6.0 (4.7; 9.6)	0.049	5.4 (4.5; 7.7)	5.3 (4.4; 6.7)	0.073
**Liver**						
ALT [U/L]	36.5 (11; 132)	41.93 (12; 263)	0.254	29.8 (8; 418)	29.92 (9; 186)	0.263
AST [U/L]	25.1 (8; 54)	29.16 (12; 140)	0.465	24.5 (11; 312)	22.33 (8; 137)	0.370
Alkaline phosphatase [U/L]	75.8 (35; 122)	83.227 (36; 131)	0.036	68.8 (30; 194)	82.71 (31; 270)	<0.001
GGT [U/L]	33.3 (9; 136)	64.99 (9; 1867)	0.431	28.5 (5; 514)	20.69 (5; 279)	<0.001
Bilirubin [µmol/L]	10.3 (3.4; 24)	8.8 (3.4; 23.9)	0.012	12.4 (3.4; 44.5)	10.2(1.7; 25.7)	0.081
Albumin [g/L]	44.2 (36.8; 50.2)	43.5 (36.1; 51.0)	0.228	43,6 (38.3; 52.8)	43.525 (37.4; 51.2)	0.529
**D** **Liver scores**						
**BARD**			0.946			0.835
0	0	0		3 (3.7)	3 (3.8)	
1	38 (46.9)	37 (46.8)		23 (28.4)	23 (29.1)	
2	10 (12.3)	15 (19)		1 (13.6)	14 (17.7)	
3	30 (37)	19 (24.1)		37 (45.7)	30 (38)	
4	3 (3.7)	8 (10.1)		7 (8.6)	9 (11.4)	
**ALBI**			0.171			0.103
1	11 (13.6)	15 (19)		6 (7.4)	11 (13.9)	
2	64 (79)	60 (75.9)		71 (87.7)	65 (82.3)	
3	0	0		1 (1.2)	0	
**NFS**			0.282			0.077
<−1.445	46 (56.8)	18 (22.8)		48 (59.3)	52 (65.8)	
>0.675	8 (9.9)	6 (7.6)		3 (3.7)	0	
**FIB-4**			1			0.303
<1.45	70 (86.4)	75 (94.9)		67 (82.7)	72 (91.1)	
>3.25	0	0		1 (1.2)	0	

## Data Availability

The data presented in this study are available on request from the corresponding author.
